# Fluorescence-Guided Probes of Aptamer-Targeted Gold Nanoparticles with Computed Tomography Imaging Accesses for *in Vivo* Tumor Resection

**DOI:** 10.1038/srep15675

**Published:** 2015-10-28

**Authors:** Cheng-Hung Li, Tsung-Rong Kuo, Hsin-Jan Su, Wei-Yun Lai, Pan-Chyr Yang, Jinn-Shiun Chen, Di-Yan Wang, Yi-Chun Wu, Chia-Chun Chen

**Affiliations:** 1 Department of Chemistry, National Taiwan Normal University, Taipei 11677, Taiwan; 2 Ph. D. Program in Graduate Institute of Nanomedicine and Medical Engineering, College of Biomedical Engineering, Taipei Medical University, Taipei 11031, Taiwan; 3 Institute of Biomedical Sciences, Academia Sinica, Taipei 10617, Taiwan; 4 Division of Pulmonary Medicine, Department of Internal Medicine, National Taiwan University Hospital, College of Medicine, Taipei 10051, Taiwan; 5 Division of Colorectal Surgery, Chang Gung Memorial Hospital, Chang Gung University, Taoyuan 33302, Taiwan; 6 Institute of Molecular and Cell Biology, National Taiwan University, Taipei, 10617, Taiwan; 7 Institute of Atomic and Molecular Science, Academia Sinica, Taipei 10617, Taiwan

## Abstract

Recent development of molecular imaging probes for fluorescence-guided surgery has shown great progresses for determining tumor margin to execute the tissue resection. Here we synthesize the fluorescent gold nanoparticles conjugated with diatrizoic acid and nucleolin-targeted AS1411 aptamer. The nanoparticle conjugates exhibit high water-solubility, good biocompatibility, visible fluorescence and strong X-ray attenuation for computed tomography (CT) contrast enhancement. The fluorescent nanoparticle conjugates are applied as a molecular contrast agent to reveal the tumor location in CL1-5 tumor-bearing mice by CT imaging. Furthermore, the orange-red fluorescence emitting from the conjugates in the CL1-5 tumor can be easily visualized by the naked eyes. After the resection, the IVIS measurements show that the fluorescence signal of the nanoparticle conjugates in the tumor is greatly enhanced in comparison to that in the controlled experiment. Our work has shown potential application of functionalized nanoparticles as a dual-function imaging agent in clinical fluorescence-guided surgery.

The development of fluorescent probes for the cellular targeting and imaging is of importance for cancer diagnosis and surgery in current clinical practices^1^,^2^,^3^,^4^,^5^,^6^,^7^,^8^,^9^. The functionalized fluorescent probe could be applied as a contrast agent for real-time visualization of the molecular edge between cancer and adjacent normal tissue, and consequent identification of the adequate tumor margin before surgery^10^,^11^,^12^. Great efforts have been made to develop new functionalized fluorescent probes using various types of inorganic nanoparticles such as quantum dots, and fluorescent silica spheres during the last decade^13^,^14^,^15^,^16^,^17^. The biomedical applications of these fluorescent nanoparticles have been mostly focused on long-term cellular imaging and tracking, but very few on cancers targeting at the whole organism level^18^,^19^,^20^. Of recent, gold nanoparticles with stable fluorescent emission have been successfully synthesized by the careful control of their size and surface modification^21^,^22^,^23^. In comparison to traditional organic fluorophores and fluorescent inorganic nanoparticles such as quantum dots for imaging applications, fluorescent gold nanoparticles could provide a high degree of flexibility in terms of functional groups for coating and targeting^24^,^25^. By the attachments on the nanoparticle surface with antibody, peptide or aptamer, the resulting fluorescent gold nanoparticle conjugates can be used as molecular imaging agents for specific cell targeting^26^,^27^,^28^. In addition, the high photostablility, nontoxicity and biocompatibility made fluorescent gold nanoparticles for long-term imaging and tracking in live cells and animals^29^,^30^.

Gold atoms can induce a strong X-ray attenuation because of the high electron density (19.32 g/cm^3^), that makes gold nanoparticles an ideal contrast agent in computed tomography (CT) in lieu of the conventional iodine-based contrast agents^31^,^32^. In the medical practices, the conventional iodine-based contrast agents usually have several drawbacks such as short imaging time, and low specificity^33^,^34^. Thus, the design of multi-functionalized gold nanoparticles as contrast agents for precise CT molecular imaging could be of significance for cancer theranostics. Gold nanoparticles attached with antibody, peptide or aptamer on their surface have been used as contrast agents in CT imaging for the identification of cancer cells^35^,^36^,^37^. For examples, the attenuation coefficient enhancement of the molecularly targeted by gold nanoparticles conjugated with UM-A9 antibodies was over 5 times than that of the identical but untargeted head and neck cancer cells or for normal cells^38^. Bombesin peptide functionalized gold nanoparticles were selectively targeted to the cancer receptors overexpressed in prostate, breast and small-cell lung carcinoma resulted in the enhancement of CT attenuation^39^. By functionalizing the surface of gold nanoparticles with the aptamer that recognizes the prostate-specific membrane antigen (PSMA), the aptamer-conjugated gold nanoparticles showed over 4-fold greater CT intensity for a targeted LNCaP cell than that of a non-targeted PC3 cell^40^. Those previous developments of gold nanoparticles as CT molecular contrast agents have brought meaningful new impacts on the cancer diagnosis by facilitating early detection and improving diagnostic accuracy.

Recently, the technique of fluorescence-guided surgery has entered the surgical theater to help operators decide which tissues need to be resected and which tissues need to be preserved during surgery^41^,^42^. These achievements could result in a great improvement on patient outcome and reduction of entire healthcare costs. Some progresses have been made on the uses of green fluorescent protein (GFP) to selectively and accurately label tumors and then to perform the resection under fluorescence guidance^43^. To the best of our knowledge, none of example has demonstrated the studies of metal or inorganic nanoparticles in *in vivo* fluorescent-guided resection. In addition to the uses in CT imaging as mentioned above, fluorescent gold nanoparticles could be further developed as optical imaging contrast agents to realize fluorescence-guided surgery for tumor treatments in medical surgery practices. However, one of the major challenges was the precise delivery of the fluorescent probes onto targeted cancer cells *in vivo* in order to clearly identify the location of tumor by CT imaging and also to provide the real time fluorescent visualization during the resection practices.

In this work, AS1411 aptamer with the specific targeting function to nucleolin was chosen. Fluorescent gold nanoparticles conjugated with diatrizoic acid and AS1411 aptamer (AS1411-DA-AuNPs) were synthesized and well characterized. The biocompatible AS1411-DA-AuNPs were evaluated for cancer-targeting molecular fluorescence imaging in a MCF-7 cell line by confocal microscopy. Moreover, their potential applications in medical imaging and fluorescence-guided surgery were investigated in mice. The AS1411-DA-AuNPs were injected into CL1-5 tumor-bearing mice via tail vein to detect the CL1-5 tumor location using animal micro-CT modality and then the fluorescence enhancement of the nanoparticles was further examined on resected CL1-5 tumor with *in vivo* imaging system (IVIS). To evaluate the targeting efficiency, the orange-red fluorescence signals coming from the AS1411-DA-AuNPs (with aptamer) in CL1-5 tumors were compared with the control experiment, which the fluorescent nanoparticles without the aptamer conjugation was applied. Our work has demonstrated a new concept that fluorescent gold nanoparticles could be applied as contrast agents for imaging and target-specific fluorescence-guided surgery in the future clinical practices.

## Results

### Characterization of AS1411-DA-AuNPs

**Figure 1:**
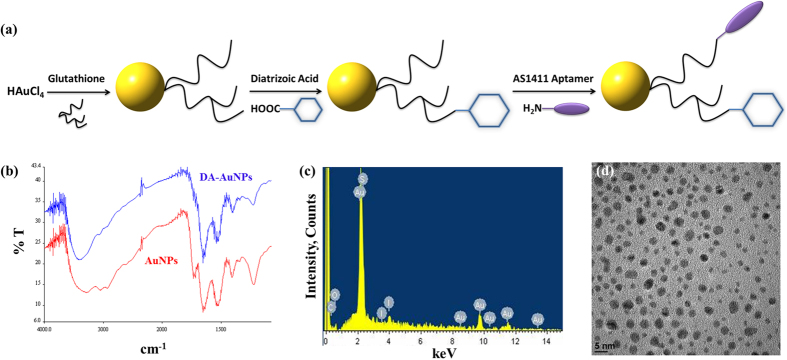
(**a**) The sequential synthetic steps of AS1411-DA-AuNPs. (**b**) The FTIR spectra of fluorescent gold nanoparticles (red) and DA-AuNPs (blue). (**c**) EDX analysis of AS1411-DA-AuNPs. (**d**) TEM image of AS1411-DA-AuNPs. The nanoparticles exhibited nearly spherical shape with the average size of 2.4
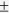
0.4 nm based on the averaged sizes of 200 nanoparticles in TEM images.

The sequential synthetic steps of AS1411-DA-AuNPs were described in Fig. 1a. In brief, the synthesis of fluorescent gold nanoparticles conjugated with diatrizoic acid and AS1411 aptamer consists of three steps. First, the fluorescent gold nanoparticles were prepared via one-pot green method. Second, the fluorescent gold nanoparticles were conjugated with commercial iodine-based contrast agent of diatrizoic acid (DA-AuNPs) to increase CT sensitivity and water solubility. Finally, DA-AuNPs were modified with AS1411 aptamer (AS1411-DA-AuNPs) using EDC activation reaction.

The conjugation of fluorescent gold nanoparticles with diatrizoic acid (DA-AuNPs) was first studied by Fourier transform infrared spectroscopy before they were conjugated with AS1411 aptamer. They exhibited the characteristic IR bands of primary amine salt (N-H stretch; 2929.88 and 3047.97 cm^−1^) and amide (N-H stretch; 3291.51 cm^−1^) as shown in Fig. 1b. Also, the characteristic N-H stretch band of amide shifted from 3291.51 to 3409.59 cm^−1^. The IR data confirmed that, after the EDC/NHS coupling reaction, the carboxyl group of diatrizoic acid was bound with primary amine of glutathione on fluorescent gold nanoparticles to form amide bond and the IR absorption of primary amine salt was disappeared. To further verify the successful conjugation of diatrizoic acid onto the surface of gold nanoparticle, the EDX analysis of DA-AuNPs were applied, and the results showed that the atomic percentages of the gold, iodine and sulfur were 14.32, 5.41, and 13.79%, respectively (Fig. 1c). The detection of iodine also implied that diatrizoic acid was successfully attached on gold nanoparticle surface. The resulting DA-AuNPs exhibited high water solubility.

Afterward, DA-AuNPs were then conjugated with nucleolin-targeted AS1411 aptamer using EDC/NHS activation reaction in aqueous solution. After the conjugation of AS1411 and DA-AuNPs, the extra AS1411 aptamer was removed thoroughly from the surface of AS1411-DA-AuNPs. The quantitative polymerase chain reaction (qPCR) of AS1411-DA-AuNPs was performed to calculate the quantity of AS1411 aptamer conjugated onto DA-AuNPs. The concentration of the aptamer of AS1411-DA-AuNPs solution was found to be 99.7 μM. The efficiency of DA-AuNPs conjugated with AS1411 aptamer was calculated to be ~66.46% via EDC/NHS activation reaction. Overall, the qPCR result suggested that the DA-AuNPs were efficiently conjugated with AS1411 aptamer. After all, the water-soluble AS1411-DA-AuNPs were examined by TEM to determine their structural properties (Fig. 1d).

### Optical Properties of AS1411-DA-AuNPs

**Figure 2:**
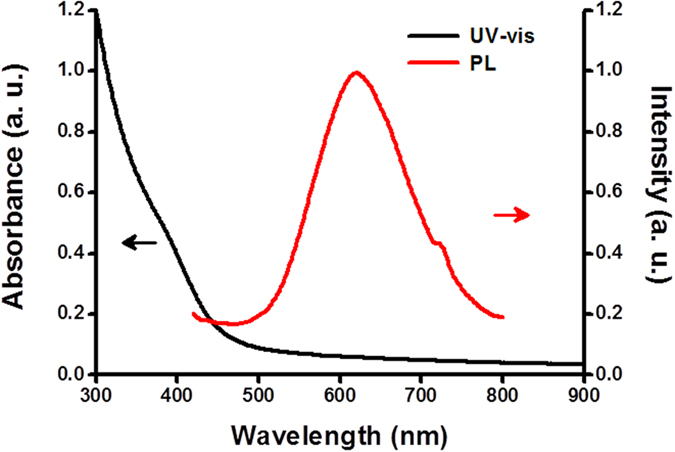
UV-vis absorption spectrum (black) and emission spectrum (red) of AS1411-DA-AuNPs. The emission spectrum of AS1411-DA-AuNPs was measured with the excitation of 400 nm wavelength. The quantum yield of orange-red emission from AS1411-DA-AuNPs was measured to be 1.1% using the fluorescence of R6G as the standard.

Figure 2 shows the UV-vis absorption and fluorescence emission spectra of AS1411-DA-AuNPs. The disappearance of the surface plasmon absorption of fluorescent AS1411-DA-AuNPs at ~520 nm could be due to the high oxidation states of extra small gold nanoparticles (~2.4 nm in diameter) and the lack of free electrons to provide free electrons to generate the coherent oscillations^44^,^45^. The fluorescence spectrum of AS1411-DA-AuNPs in Fig. 2 showed clear orange-red emission with the maximum at 620 nm. Several studies suggested that the emission of AS1411-DA-AuNPs could be induced from ligand-metal charge transfer on thiolate capped gold nanoparticles (~2 nm), which the electrons were transferred from the sulfur atom of surface glutathione to the Au core^46^,^47^,^48^. Notably, the distinguishable observable orange-red color of high water-soluble AS1411-DA-AuNPs was readily seen by naked eyes under irradiation of a hand-held long-wave UV lamp. This characteristic makes AS1411-DA-AuNPs a candidate for fluorescent molecular imaging.

### Cell Viability Assays of AS1411-DA-AuNPs

**Figure 3:**
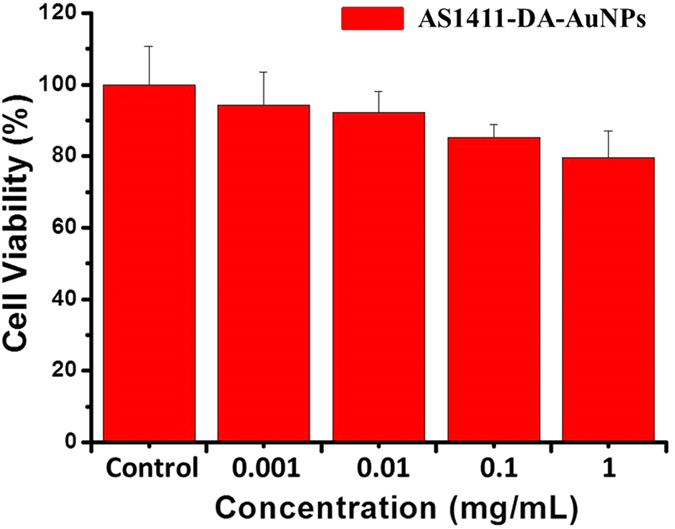
Cytotoxicity evaluated by MTT assay in the range of 1-1000 μg/mL of AS1411-DA-AuNPs. The MCF-7 cells were incubated with AS1411-DA-AuNPs solution for 24 h.

The cytotoxicity measurements were evaluated in a MCF-7 cell line by MTT assay to reveal the biocompability of AS1411-DA-AuNPs^49^. The assay was performed over a dosage range of 10^−3^ ~ 1 mg/mL of AS1411-DA-AuNPs solutions. In Fig. 3, the MTT assay showed no significant cytotoxic response (the cell viability >80%) detected even at the concentration of 1 mg/mL, which was much higher than that usually used for live-cell imaging studies by confocal microscopy. This study indicated that the AS1411-DA-AuNPs have shown very low cytotoxicity. We therefore explore their applications as a molecular contrast agent.

### Cell Targeting and Imaging

**Figure 4:**
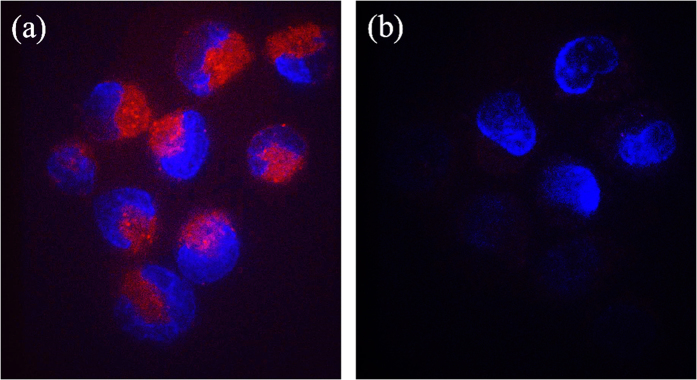
Two-dimensional confocal microscopic images of CL1-5 cells (a) incubated with AS1411-DA-AuNPs and (b) incubated with DA-AuNPs. The red and blue pseudocolors represent the fluorescent signal of AS1411-DA-AuNPs and the nucleus (stained with Hoechst 33258), respectively.

To study the potential of the nanoparticles as fluorescent probes for biomedical imaging, the CL1-5 cells were separately incubated with DA-AuNPs (without aptamer attached) and AS1411-DA-AuNPs (with aptamer attached) for the comparison. After 30 min, the cells of both samples were compared under a confocal microscope. As shown in Fig. 4a, the fluorescence of AS1411-DA-AuNPs (red pseudocolor) was clearly observed with high signal-to-background ratio in the CL1-5 cells incubated with AS1411-DA-AuNPs. In contrast, under the same imaging condition, no detectable fluorescence of DA-AuNPs was observed in the CL1-5 cells incubated with DA-AuNPs (Fig. 4b). The result showed that the fluorescent AS1411-DA-AuNPs, but not DA-AuNPs, entered efficiently into the CL1-5 cells. Thus, the AS1411 aptamer of AS1411-DA-AuNPs leaded to the specific binding to nucleolin highly expressed CL1-5 cells in the plasma membrane.

Overall, our experiments clearly demonstrated that AS1411-DA-AuNPs exhibited the capability as fluorescent probes for cancer targeting.

### *In Vitro* CT Imaging of AS1411-DA-AuNPs

**Figure 5:**
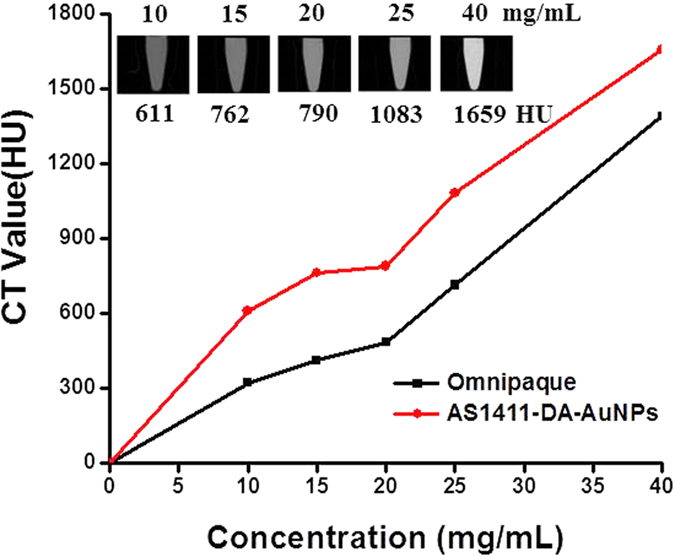
The CT values of AS1411-DA-AuNPs and Iohexol (Omnipaque). The insets showed the CT images, the respective weight concentrations of AS1411-DA-AuNPs (above the image), and the respective CT values (below the image).

We first examined the CT values of fluorescent gold nanoparticles before and after the conjugation with diatrizoic acid. The CT signal intensity of DA-AuNPs was increased about 11% in HU value to that of the fluorescent gold nanoparticles at the same gold weight concentration. Next, we explored the possibility of using AS1411-DA-AuNPs as a contrast agent in CT imaging. The CT signal intensity of AS1411-DA-AuNPs was measured at the weight concentrations in range of 10 to 40 mg/mL. The CT signal intensity in Fig. 5 revealed positive contrast enhancement in a dose-dependent manner. Also, when the weight concentration changed from 10 to 40 mg/mL, the signal intensity of AS1411-DA-AuNPs exhibited much higher CT signal intensity than that of the commercially available iodine-containing agents (Omnipaque, Ge Healthcare) at the same concentration. The strong CT signal intensity of AS1411-DA-AuNPs was mainly generated from the high X-ray absorption coefficient of gold and iodine (X-ray absorption coefficient at 100 keV, Au: 5.16 cm^2^/g; I: 1.94 cm^2^/g).

### *In Vivo* CT Imaging to Detect CL1-5 Tumor Location

**Figure 6:**
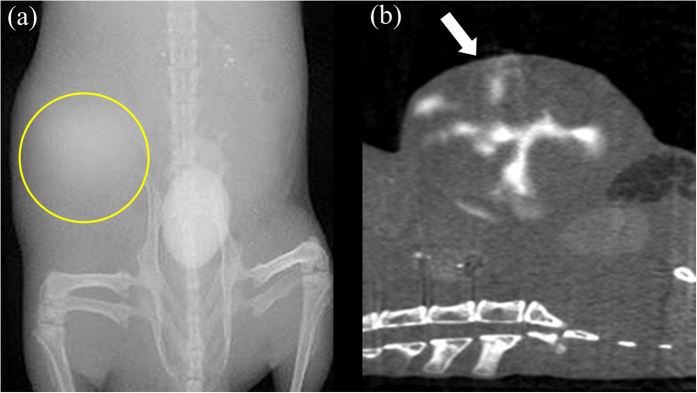
(**a**) The CT image of the CL1-5 tumor-bearing mouse was taken at 30 min post injection of AS1411-DA-AuNPs. The location of CL1-5 tumor was marked by yellow circle. (**b**) The axial CT image of the CL1-5 tumor. The location of CL1-5 tumor was marked by white arrow.

The *in vivo* molecular imaging was studied on the CL1-5 tumor-bearing mice. The target-specific AS1411-DA-AuNPs (370 mg/mL, 100 *μ*L) were injected through tail vein into the CL1-5 tumor-bearing mice. The CT imaging was performed in the mice using animal micro-CT modality (SkyScan1076, Bruker) to identify the location of tumor. The CT images were acquired before and after injection of the AS1411-DA-AuNPs. At 30 min post injection, a distinct 106% more contrast enhancement of the CL1-5 tumor targeted with AS1411-DA-AuNPs was measured in CL1-5 tumor-bearing mouse (marked by yellow circle in Fig. 6a). The axial CT image of the CL1-5 tumor was also shown in Fig. 6b. The CT image showed clearly that AS1411-DA-AuNPs were predominantly accumulated in the bladder, which later excreted out of the mice rapidly. The animal experiments showed that the AS1411-DA-AuNPs were able to be the molecular contrast agent to detect the tumor location in mice using CT modality. After the detection of CL1-5 tumor location by CT imaging, the tumor-bearing mice were sacrificed. The CL1-5 tumor targeted with AS1411-DA-AuNPs in the tumor-bearing mice was further examined under ultraviolet light irradiation to demonstrate the applications as intraoperative fluorescent imaging probes in guided surgery.

### AS1411-DA-AuNPs for Intraoperative Fluorescence-Guided Resection in Mouse Model

**Figure 7:**
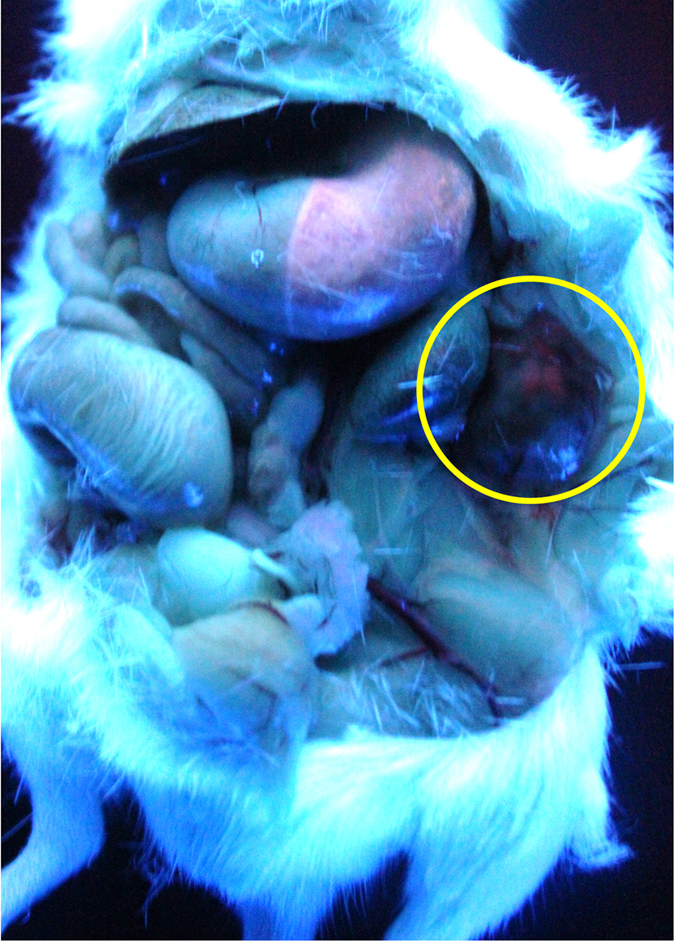
The fluorescence image of CL1-5 tumor-bearing mouse at 30 min post injection of AS1411-DA-AuNPs. The yellow circle points out the location of the CL1-5 tumor.

The fluorescence imaging was performed under a handy ultraviolet light (4W) in Fig. 7. The orange-red fluorescence coming from AS1411-DA-AuNPs was readily observed in the CL1-5 tumor (marked by yellow circle) and can be seen by naked eyes. The fluorescence of AS1411-DA-AuNPs was further used for the identification of the CL1-5 tumor margin. The tissue with orange-red fluorescence was resected and the normal tissue without orange-red fluorescence was preserved in the tumor-bearing mouse. Thus, the CL1-5 tumor targeted with AS1411-DA-AuNPs was successfully taken out by intraoperative fluorescence-guided resection. This result has demonstrated the great potentials of using AS1411-DA-AuNPs as a molecular imaging probe in fluorescent-guided surgery. In comparison to most currently used organic molecules, AS1411-DA-AuNPs are able to provide long-term imaging times, high photostability, multiple imaging functions and feasible surface modifications with specific-targeted molecules. However, in order to quantitatively evaluate the specific targeting efficiency of AS1411-DA-AuNPs, the further experiments on fluorescent signal measurements by IVIS were performed next.

### Targeting Enhancement Analysis of AS1411-DA-AuNPs

**Figure 8:**
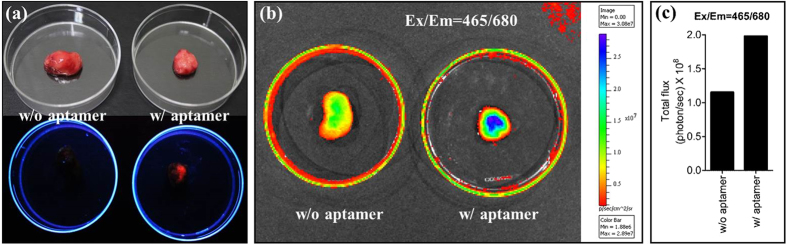
(**a**) CL1-5 tumors under white light (top panel) and ultraviolet light (bottom panel). CL1-5 tumors incubated with DA-AuNPs (left column) and AS1411-DA-AuNPs (right column). (**b**) The IVIS images of CL1-5 tumors incubated with DA-AuNPs (left) and AS1411-DA-AuNPs (right). The color scale is an indication of the fluorescent intensity from red (low) to blue (high). (**c**) The total photon fluxes calculated from CL1-5 tumors shown in Fig. 8b.

Two groups of CL1-5 tumor-bearing mice, A and B, were separately injected via tail vein with AS1411-DA-AuNPs (with AS1411 aptamer) and DA-AuNPs (without aptamer). At 30 min post-injection, the mouse A and B were sacrificed and then the CL1-5 tumors in the mouse A and B were taken out. Figure 8a (top panel) showed that the CL1-5 tumors were observed under white light. The color and size of both CL1-5 tumors were similar. However, under excitation by ultraviolet light, the CL1-5 tumor of mouse A injected with AS1411-DA-AuNPs revealed orange-red fluorescence as shown in Fig. 8a (bottom panel), whereas no red fluorescence was observed in the CL1-5tumor of mouse B injected with DA-AuNPs. Those images in Fig. 8a suggested that the AS1411-DA-AuNPs have been specifically targeted into the nucleolin highly expressed CL1-5 tumor, and the fluorescent nanoparticles could be potentially used as intraoperative fluorescence imaging probes during guided resection.

To further quantitatively analyze the targeting efficiency, IVIS imaging was utilized to compare the fluorescent intensity of the CL1-5 tumors of mouse A and mouse B. The IVIS image in Fig. 8b (right) demonstrated that the strong fluorescence was observed in the CL1-5 tumor targeted with AS1411-DA-AuNPs of mouse A. The fluorescent intensities, which were calculated with the IVIS image, ranged from 1.88 × 10^6^ ~ 2.89 × 10^7^ p/sec/cm^2^/sr. However, with the control experiment of DA-AuNPs injection, the fluorescence in the CL1-5 tumor of mouse B was not detected at the background level. Furthermore, the total photo flux in CL1-5 tumors was measured to evaluate the enhancements of fluorescence with mouse A and mouse B (Fig. 8c). The total photon fluxes in the CL1-5 tumors are 1.98 × 10^8^ and 1.15 × 10^8^ p/sec/cm^2^/sr with mouse A and mouse B, respectively. The fluorescent enhancement of CL1-5 tumor with targeting AS1411-DA-AuNPs injection of mouse A was 172% in comparison to non-targeting DA-AuNPs injection of mouse B. Overall, the *in vivo* experiments in Fig. 8 have showed that the AS1411 aptamer conjugated with fluorescent gold nanoparticles can be used for selectively targeting nucleolin highly expressed CL1-5 tumor, and the strong fluorescence of AS1411-DA-AuNPs was sufficient to contrast the tumor lesions.

## Discussion

Our *in vivo* studies on CL1-5 tumor-bearing mice showed that the AS1411-DA-AuNPs have applied as a molecular contrast agent to detect the CL1-5 tumor location by CT imaging. The orange-red fluorescence emitting from AS1411-DA-AuNPs in CL1-5 tumor has been easily seen by the naked eyes in the tumor-bearing mice. And, the CL1-5 tumor was successfully resected from the mouse under visible fluorescence guiding. With the target-specific guiding of florescent gold nanoparticle (AS1411-DA-AuNPs), the fluorescence signal of resected CL1-5 tumor was enhanced by 172% compared to that of DA-AuNPs by IVIS measurements.

Overall, our experiments have established a simple CT/fluorescent imaging platform using gold nanoparticle conjugates for tumor imaging and resecting in a mice model. The platform has demonstrated several advantages in comparison to other molecular imaging systems. First, besides the lung cancer in a mouse model studied in this work, our platform could be also applicable in other tumor systems. Since the synthetic procedures of this platform were relatively simple, a fluorescent molecular probe could be easily prepared by the conjugation of DA-AuNPs with specific targeted aptamers (antibodies or peptides) to perform molecular imaging. Secondly, AS1411-DA-AuNPs exhibit safe and simple chemical properties to avoid the complexities of tumor-selective gene delivery in comparison to the green fluorescent protein that was recently developed as the fluorescent imaging agent in the field of fluorescence-guided surgery. Third, in our platform, the CT and fluorescence images were simultaneously obtained, allowing us to double check the targeted tumor information such as the location, shape and size. Therefore, much comprehensive diagnostic information could be provided using AS1411-DA-AuNPs as a contrast agent, and the resection of the tumor could become feasible under fluorescence-guided surgery. Finally, the platform has offered a simple method to determine tumor margin based on the images from IVIS measurements after the tumor resection. However, we understand that, in our experiment, the correlation between fluorescence intensity and the amount of tumor cells should be further analyzed quantitatively. In the future, other analytical methods for *in vivo* tumor cell determination should be applied to make a comparison with the results obtained from IVIS measurements. Thus, the florescent intensity from IVIS measurements could be transformed to be much quantitative information in order to create a clear guideline on the determination of tumor margins. Although it could be a long way to go for preclinical or even clinical tests, we expect eventually that our platform based on fluorescent gold nanoparticle conjugates will become a powerful tool in the field of fluorescence-guided surgery.

## Methods

### Materials

Hydrogen tetrachloroaurate(III) trihydrate (HAuCl_4_·3H_2_O, Alfa Aesar, 99.99%), L-glutathione reduced (C_10_H_17_N_3_O_6_S, Sigma-Aldrich, ≥98.0%), N-(3-dimethylaminopropyl)-N’-ethylcarbodiimide hydrochloride (C_8_H_17_N_3_·HCl, Sigma-Aldrich, ≥98.0% ), N-hydroxysuccinimide (C_4_H_5_NO_3_, Alfa Aesar, ≥98.0%), meglumine diatrizoate (C_7_H_17_NO_5_·C_11_H_9_I_3_N_2_O_4_, Sigma-Aldrich), MES hydrate (C_6_H_13_NO_4_S·xH_2_O, Sigma-Aldrich, ≥99.5%), phosphate-buffered saline (PBS, 1X, pH 7.4, GIBCO) and AS1411 aptamer (5′-TTG GTG GTG GTG GTT GTG GTG GTG GTG G-3′, Sigma-Aldrich).

### Preparation of Fluorescent Gold Nanoparticles

The fluorescent gold nanoparticles were prepared by a one-pot green method with some modifications^50^. Briefly, 15 mL of HAuCl_4_ aqueous solution (1% w/w) was added to 15 mL of 25 mM L-glutathione aqueous solution under vigorous stirring. The color of HAuCl_4_ and L-glutathione solution changed from transparent to dark brown and finally became transparent. The HAuCl_4_ and L-glutathione solution was then heated to 40 °C with vigorous stirring in the dark environment for 3 ~ 5 days. After 3 ~ 5 days, the fluorescent gold nanoparticle solution was obtained. The solution was subsequently purified by centrifuging at 15000 rpm for 5 min. The supernatant solution was kept after removal of the particles in the bottom. The supernatant was further precipitated by adding ethanol (the volume ratio between supernatant and ethanol was 1:2) and the yellow cloudy mixture was formed. Afterward, the mixture was centrifuged at 18000 rpm for 10 min. A precipitate of fluorescent gold nanoparticles was formed at the bottom of the centrifuge tube. After the removal of supernatant solution in the centrifuge tube, the precipitate of fluorescent gold nanoparticles was redispersed in deionized water with 80 mg N-methyl-D-glucamine by sonication. The purified fluorescent gold nanoparticles were stored at 4 °C in the dark environment for the following experiments. The fluorescent gold nanoparticle solution was stable more than 6 months.

### Conjugation of Fluorescent Gold Nanoparticles with Meglumine Diatrizoate

0.5 M 1-ethyl-3-(3-dimethylaminopropyl) carbodiimide (EDC) (1.151 g) and 0.5 M N-hydroxysuccinimide (NHS) (1.553 g) were prepared in 20 mL 2-(*N*-morpholino) ethanesulfonic acid (MES) (0.1 M), respectively. First, 302.4 mg meglumine diatrizoate was dissolved in 10 mL deionized water. Second, 750 μL of 0.5 M EDC and NHS were sequentially added in meglumine diatrizoate solution. The mixture was stirred at room temperature for 1 h. Third, fluorescent gold nanoparticle solution was added in the mixture and then was stirred at room temperature for 12 h. The solution of fluorescent gold nanoparticles conjugated with diatrizoic acid (DA-AuNPs) was washed with 20 mL ethanol and then centrifuged at 18000 rpm for 10 min. The pellet of the DA-AuNPs was redispersed in deionized water. Finally, the DA-AuNPs were collected and filtrated by 0.22 μm syringe filter (Millipore MILLEX® MCE membrane) to remove larger nanoparticles.

### Synthesis of DA-AuNPs Conjugated with Aptamer

The aqueous solution of DA-AuNPs was dried under vacuum. The dried DA-AuNPs were dispersed in PBS buffer solution (87.5 mg/mL) for the following experiments. 300 μL of 100 μM AS1411 aptamer was added in a 20 mL flask^51^. And then 30 μL of 5 mM EDC and NHS were sequentially added in the flask. 200 μL of 87.5 mg/mL DA-AuNPs was also added in the flask. At this time, the flask was added with 10 mL deionized water and the reactants were stirred at 4 °C for 2 h. After 2 h reaction, the AS1411-DA-AuNPs solution was added with the same volume ethanol and then centrifuged at 18000 rpm for 5 min. The supernatant solution was slowly removed without disturbing the precipitate. The precipitate was dissolved in deionized water. Afterward, the AS1411-DA-AuNPs aqueous solution was dried under vacuum. The dried AS1411-DA-AuNPs were redispersed in PBS buffer solution and finally stored at 4°C for the following experiments.

### Characterization Techniques

Instruments of transmission electron microscope (TEM) and X-ray energy-dispersive spectroscopy (EDX) were performed on a Philips/FEI Tecnai 20 G2 S-Twin transmission electron microscope. A small amount of AS1411-DA-AuNPs was dispersed in deionized water by sonicator. A drop of AS1411-DA-AuNPs solution was placed on an amorphous carbon membrane supported by a copper grid. The copper grid was characterized by TEM and EDX. The ligand attachment of fluorescent gold nanoparticles was characterized by FTIR spectrophotometer (JASCO 200E FT-IR) with the resolution of 2 cm^−1^. UV-vis absorption and fluorescence spectroscopy was measured by using PerkinElmer HP-8453 and AMINCO Bowman Series 2 spectrometers, respectively, with the resolution of 1 nm.

### Cell Viability Assays of AS1411-DA-AuNPs by MCF-7 Cells

Dulbecco’s Modified Eagle Medium (DMEM) containing 3.7 g/L sodium bicarbonate, 1% penicillin, and 10% fetal bovine serum was used to maintain human breast cancer cell line MCF-7. The MCF-7 cell culture was placed in a 37 °C humidified atmosphere with 5% CO_2_, and DMEM was refreshed every 3 days. For cell viability assays of AS1411-DA-AuNPs, MCF-7 cells were first seeded onto 96-well plates at a density of 6000 cells per well. After 100 μL of DMEM was added to each well, the 96-well plates were returned to incubators for cell attachment about 12 h. Following cell attachment, each well was added with another 100 μL of AS1411-DA-AuNPs solution for 24 h. After the removal of supernatant, the MCF-7 cells were then treated with 20 μL of MTT (5 mg/mL in PBS) and then incubated for 4 h. Finally, the medium was eliminated, and the cells were lysed with DMSO (100 μL). The absorbance of the purple formazan was recorded at 570 nm by Nanodrop spectrophotometer.

### Confocal Imaging of AS1411-DA-AuNPs Targeted to CL1-5 Cells

The human lung adenocarcinoma cell line CL1-5 was cultured in 25 cm^2^ flask with RPMI-1640 medium containing 10% (v/v) fetal bovine serum at 37 °C under 5% CO_2_ environment. After several days, the CL1-5 cells were put on quartz bottom dishes and incubated for 24 h. Before the incubation with the fluorescent nanoparticle conjugates, the CL1-5 cells were washed with PBS solution (pH = 7.4). For the comparison of the specific targeting efficiency of the fluorescent nanoparticle conjugates with and without AS1411 aptamer on their surface, AS1411-DA-AuNPs and DA-AuNPS (100 ng/mL) were respectively incubated with CL1-5 cells at 37 °C for 30 min. Afterward, the CL1-5 cells were washed with PBS buffer solution for three times and then the CL1-5 cells were further fixed with 4% paraformaldehyde for 10 min at the room temperature. Hoechst 33258 (Invitrogen, 0.5 μg/mL) was treated for 5 min to stain the CL1-5 cell nucleus. Confocal fluorescence microscopy was carried out with an Ultra-View RS confocal system (Perkin Elmer, Wellesley, MA).

### CT Imaging of AS1411-DA-AuNPs in Mice

All the animal studies were performed according to the protocols approved by the Laboratory Animal Center, Academia Sinica. 8-week old NOD-SCID mice (BioLasco, Taiwan) were subcutaneously inoculated with 2 × 10^6^ CL1-5 cells by using a 23-gauge needle. The length (L) and width (W) of tumors were measured with calipers and the tumor volumes were calculated as LW^2^/2. After 20 days, tumor volumes were about 400 mm^3^. Mice were randomly divided into two groups: mouse A (*n* = 3) and mouse B (*n* = 3). AS1411-DA-AuNPs and DA-AuNPs (100 μL, 370 mg/mL) were intravenously injected into mouse A and mouse B, respectively. The CT signal intensity was measured and calculated by micro-CT (SkyScan1076, Bruker) at different time intervals (0, 0.5, 2, 4 and 24 h) after the injection. The measurement of CT signal before injection was used as baseline for HU value calculation.

## Additional Information

**How to cite this article**: Li, C.-H. *et al.* Fluorescence-Guided Probes of Aptamer-Targeted Gold Nanoparticles with Computed Tomography Imaging Accesses for *in Vivo* Tumor Resection. *Sci. Rep.* **5**, 15675; doi: 10.1038/srep15675 (2015).
